# Galassi type III arachnoid cyst presenting as a migraine of weariness

**DOI:** 10.1002/ccr3.6891

**Published:** 2023-01-20

**Authors:** Ahtesham Khizar, Waleed Shahzad, Pradhumna Kumar Yadav

**Affiliations:** ^1^ Pakistan Institute of Medical Sciences Islamabad Pakistan; ^2^ Janaki Health Care and Research Center Janakpur Nepal

**Keywords:** arachnoid cysts, headache, migraine

## Abstract

A 24‐year‐old right‐handed male presented with a 4‐month history of migraine of weariness. Typical accompanying symptoms with migraine like nausea, vomiting, photophobia and aura were not present. Non‐contrast CT brain revealed a left sided large frontotemporoparietal (Galassi Type III) arachnoid cyst. The patient underwent a cystoperitoneal shunt.

Arachnoid cysts are benign lesions which account for 1% of all non‐traumatic intracranial masses. Their mechanism of formation is not completely understood and the natural case history is not well documented. Several patients may have no symptoms throughout their lives, while others may become symptomatic many years after the cyst is discovered.^1^ The middle fossa or the sylvian fissure is the most prevalent site for arachnoid cysts (50%). There are a variety of surgical treatments available, and they all have a pretty comparable prognosis in terms of recurrence and morbidity. The many surgical procedures available include cystoperitoneal shunt, cyst marsupialization, cyst fenestration with the surrounding cistern either endoscopically or microsurgically, and arachnoidoplasty.^2^

A 24‐year‐old right‐handed man with a 4 month history of migraine of weariness was referred to our outpatient by a neurologist. Migraine of weariness is characterized by fatigue and exhaustion to the point where the migraineur may feel nearly unable to respond to anything. He used to have extreme fatigue and exhaustion alongside unilateral headache. Nausea, vomiting, photophobia, and aura, which are common migraine symptoms, were not present. Neurological examination was unremarkable. Baseline investigations were normal. A left sided large frontotemporoparietal (Galassi Type III) arachnoid cyst was discovered on a non‐contrast CT scan of the brain (Figure 1). A cystoperitoneal shunt was performed on the patient. Postoperatively his symptoms were improved.

**FIGURE 1:**
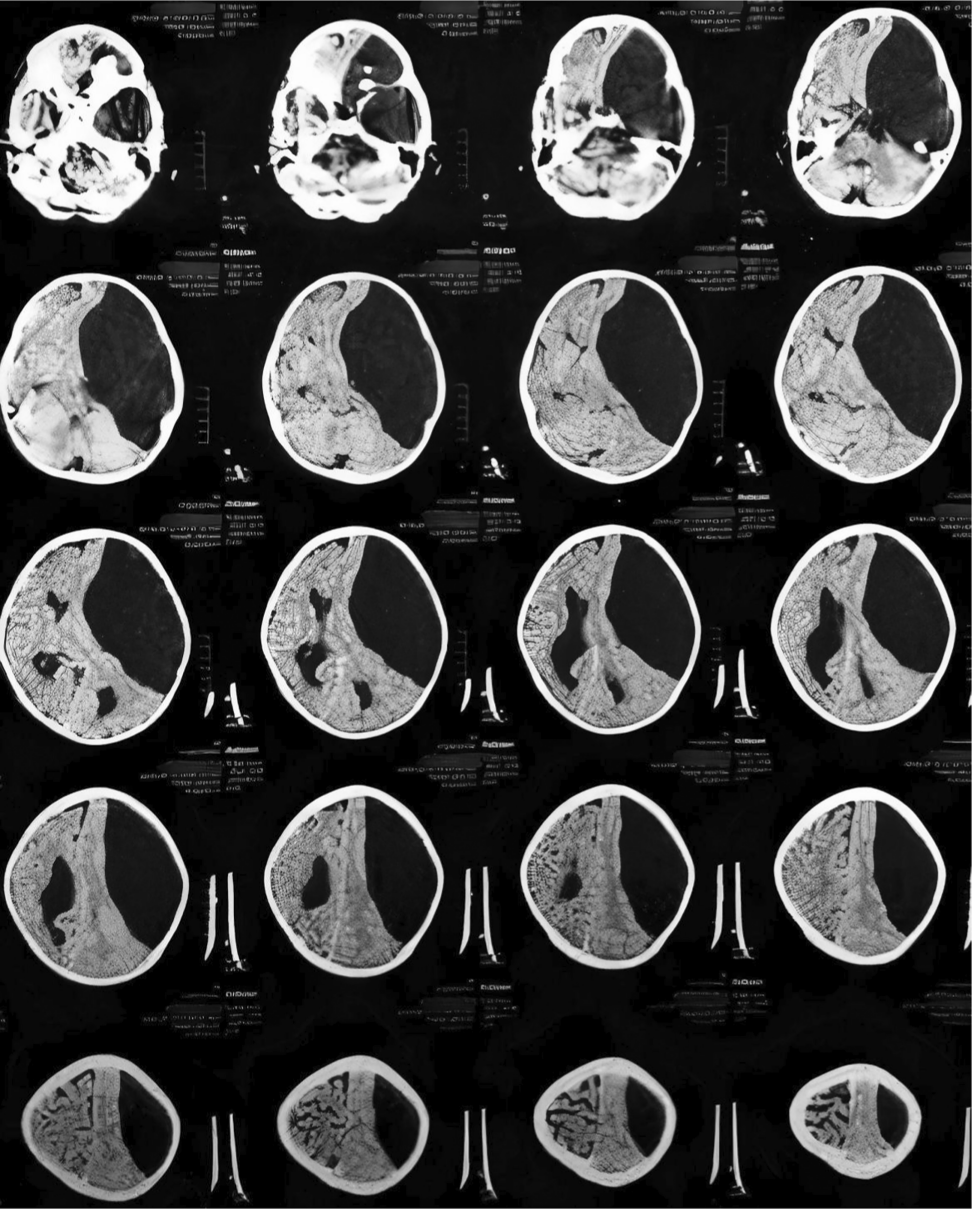
Non‐contrast computed tomography (CT) brain showing left sided large Frontotemporoparietal (Galassi Type III) Arachnoid cyst. A midline shift secondary to the mass effect of the cyst can be noted.

## AUTHOR CONTRIBUTIONS

**Ahtesham Khizar:** Conceptualization; methodology; writing – original draft; writing – review and editing. **Waleed Shahzad:** Formal analysis; investigation; resources; software; visualization. **Pradhumna Kumar Yadav:** Formal analysis; methodology; supervision; visualization.

## CONFLICT OF INTEREST

The authors declare no conflict of interest.

## ETHICS STATEMENT

Not applicable.

## CONSENT

Written informed consent was obtained from the patient to publish this report in accordance with the journal's patient consent policy.

## Data Availability

All the data is available within the article.

## References

[1] Li X , Fan Y , Li L , et al. Huge frontal‐temporal lobe arachnoid cyst presenting as an weariness migraine. J Craniofac Surg. 2016;27(3):e253‐e255. doi:10.1097/SCS.0000000000002469 26999696

[2] Magar BT , Thapa DK , Tamrakat K , Nepal PR . Management of Galassi type 3 arachnoid cyst‐a case report. Eastern Green Neurosurg. 2019;1(1):17‐20. doi:10.3126/egn.v1i1.25502

